# Reduced Oxygen Uptake Efficiency Slope in Patients with Cardiac Sarcoidosis

**DOI:** 10.1371/journal.pone.0102333

**Published:** 2014-07-16

**Authors:** Wilhelm Ammenwerth, Henrik Wurps, Mark A. Klemens, Catharina Crolow, Jeanette Schulz-Menger, Nicolas Schönfeld, Roland C. Bittner, Torsten T. Bauer

**Affiliations:** 1 Department of Pneumology, Lungenklinik Heckeshorn, HELIOS Klinikum Emil von Behring, Berlin, Germany; 2 Institute of Radiology and Nuclear Medicine, HELIOS Klinikum Emil von Behring, Berlin, Germany; 3 WG Cardiac MRI ECRC a joint institution of the MDC and Charité Medical University Berlin and HELIOS Klinikum Berlin-Buch Department of Cardiology and Nephrology, Berlin, Germany; Scuola Superiore Sant'Anna, Italy

## Abstract

**Background:**

The non-invasive diagnosis of cardiac sarcoidosis (CS) is difficult. Cardiovascular magnetic resonance (CMR) has become a very valuable diagnostic tool in patients with suspected CS, but usually a combination of different tests is used. Oxygen uptake efficiency slope (OUES) is a parameter of cardiopulmonary exercise testing (CPET), which is used as an indicator for cardiovascular impairment. We investigated the predictive value of OUES for the diagnosis of myocardial involvement in sarcoid patients.

**Methods:**

Retrospectively 37 consecutive patients (44.9±13.8 years) with histologically confirmed sarcoidosis and clinical suspicion of heart involvement underwent noninvasive diagnostic testing including CMR. CS was diagnosed according to the guidelines from the Japanese Society of Sarcoidosis and other Granulomatous Disorders with additional consideration of CMR findings. Furthermore, CPET with calculation of predicted OUES according to equations by Hollenberg et al. was carried out.

**Results:**

Patients with CS (11/37; 30%) had a worse cardiovascular response to exercise. OUES was significantly lower in CS-group compared to non-CS-group (59.3±19.1 vs 88.0±15.4%pred., *p<0.0001*). ROC curve method identified 70%pred. as the OUES cut-off point, which maximized sensitivity and specificity for detection of CS (96% sensitivity, 82% specificity, 89% overall accuracy). OUES <70%pred. was the single best predictor of CS (Odds ratio: 100.43, 95% CI: 1.99 to 5064, *p<0.001*) even in multivariate analyses.

**Conclusion:**

OUES assessed in CPET may be helpful in identifying patient with cardiac involvement of sarcoidosis. Patient selection for CMR may be assisted by CPET findings in patients with sarcoidosis.

## Introduction

Sarcoidosis is a multisystemic disease of unknown origin in usually young patients. Lung (90%), liver (30–40%), skin (20–35%), eyes (20–30%), musculoskeletal (2–38%) and nervous system (1–5%) are the organs most commonly affected. Cardiac involvement is associated with a poor prognosis and is extremely difficult to detect. Although cardiac involvement is present in 5–30% of patients with systemic sarcoidosis, only about 5% of these patients are symptomatic [1]. The absence of clinical symptoms does not rule out cardiac sarcoidosis, which is documented by the high rate of unexpected cardiac sarcoidosis in postmortem studies [2], [3]. The clinical presentation of sarcoid heart disease includes congestive heart failure, arrhythmias, conduction disturbances, pericardial effusion and also sudden death [4]. The diagnosis can be made by several diagnostic approaches [5] but is confirmed by either noncaseating granulomas in myocardial biopsy or diagnosed by evidence of granulomas in any extracardiac tissue in association with cardiac abnormalities unexplained by other causes. Cardiac sarcoidosis is much more common in the Japanese population compared to other regions and is e.g. the leading cause of death from sarcoidosis in Japan. Therefore, the Japanese Ministry of Health and Welfare published in 1993 guidelines for diagnosing cardiac sarcoidosis [6], which were revised in 2006 by the Japanese Society of Sarcoidosis and other Granulomatous Disorders (Table 1) [7]. These guidelines combine the results of various diagnostic tests and provide an useful framework, particularly for patients with proven systemic sarcoidosis in whom cardiac involvement is suspected. However, cardiovascular magnetic resonance imaging (CMR) has become a diagnostic tool of growing impact for noninvasive assessment of myocardial inflammation in patients with suspected cardiac sarcoidosis[8]–[11]. Thus, CMR was included in the revised Japanese guidelines, but is currently an expensive method with usually limited access.

**Table 1:** Summary of the 2006 revised guidelines of diagnosing cardiac sarcoidosis of the Japanese Society of Sarcoidosis and Other Granulomatous [7].

**I. Histologic diagnosis group:**
Endomyocardial biopsy demonstrates noncaseating epithelioid granulomata.
**II. Clinical diagnosis group:**
Cardiac sarcoidosis is diagnosed in the absence of an endomyocardial biopsy specimen when extracardiac sarcoidosis has been proven and a combination of major or minor diagnostic criteria has been satisfied as follows:
- More than 2 of 4 major criteria are satisfied, OR
- 1 of the 4 major criteria and 2 or more of the minor criteria are satisfied
**Major criteria**
(1) Advanced AV block
(2) Basal thinning of the ventricular septum
(3) Positive cardiac gallium uptake
(4) Left ventricular ejection fraction less than 50%
**Minor criteria**
(1) Abnormal electrocardiogram findings including ventricular tachycardia (VT), multifocal frequent premature ventricular contractions (PVC), complete right bundle branch block (RBBB), pathologic Q waves or abnormal axis deviation
(2) Abnormal regional wall motion, ventricular aneurysm or unexplained increase in wall thickness
(3) Perfusion defects detected by myocardial scintigraphy
(4) Delayed gadolinium enhancement of the myocardium (LGE) on CMR
(5) Interstitial fibrosis or monocyte infiltration greater than moderate grade by endomyocardial biopsy

RBBB  =  complete right bundle branch block. VT  =  ventricular tachycardia. PVC  =  premature ventricular contraction.

Cardiopulmonary exercise testing (CPET) is a widely accessible method for measuring physical performance including cardiovascular function. The oxygen uptake efficiency slope (OUES) is a novel parameter derived from oxygen uptake (VO_2_) and minute ventilation (VE)[12]. OUES measures cardiopulmonary functional reserve and may serve for screening of cardiovascular dysfunction[13]. To our knowledge, this tool has never been applied to patients with sarcoidosis in order to assess cardiac function. The aim of this investigation was to find out whether the OUES is a clinically useful predictor of cardiac involvement in patients with biopsy specimen-proven sarcoidosis.

## Methods

The patient records was anonymized and de-identified prior to analysis. The study was approved by the ethics committee of the Charité-University Medicine Berlin, Germany. We studied retrospectively 37 consecutive patients (44.9±13.8 years) with biopsy proven sarcoidosis and clinical evidence for cardiac involvement. Studies on the issue of cardiac involvement included electrocardiography (ECG), transthoracic echocardiography and Holter-ECG. Further investigation in suspected cardiac sarcoidosis was cardiovascular magnetic resonance (CMR), in case of unclear findings a coronary angiography was performed. In brief, cardiac sarcoidosis was diagnosed according to revised guidelines from the Japanese Society of Sarcoidosis and other Granulomatous Disorders (Table 1) [7] and additional consideration of clearly defined CMR-criteria in 11 patients [14]. The study was approved by the ethics committee of the Charité-University Medicine Berlin, Germany.

CMR Studies were performed using a 1.5-T MRI-Scanner (Achieva, Philips Medical Systems, Amsterdam', The Netherlands) with a cardiac-dedicated, five element phased array coil (SENSE), respectively with a body coil at sequences for semiquantitative analysis of myocardial inflammation following the recommendations as described by an International Consensus Group on CMR Diagnosis of Myocarditis [14]. We examined cardiac function (i.e., left ventricular ejection fraction  =  LVEF, wall motion) with a steady state free precession gradient echo cine sequence (three long axis images and at least three short axis images).

T2-weighted triple-inversion-recovery (STIR) images were acquired during the patient's breath hold by using the body coil in short axis. The following parameters were used: 15-mm section thickness, TE-time of 61-msec. Evidence for regional edema, or a signal intensity ratio of ≥2.0 (signal intensity normalized to skeletal muscle in the same slice), renders T2 findings positive.

EGE was performed before and after the intravenous injection of 0.2 ml of Gadoteridol (ProHance®, Bracco IMAGING Deutschland GmbH, Konstanz, Germany) per kilogram of body weight for acquisition of transverse sections. The following parameters were used: repetition time of one R-R interval, TE-time of 14-msec, 15-mm section thickness. An additional T1- weighted fast spin-echo saturation section was positioned across the atria to reduce the blood-signal. Quantitative evaluation of the signal enhancement (skeletal-muscle normalized myocardial enhancement ratio of ≥4.0 or an absolute enhancement of ≥45%) has been considered as a positive criterion.

LGE images were acquired, on average, 10 minutes after the intravenous administration of a second bolus of Gadoteridol (0.2 ml/kg) by using a look-locker-sequence to clarify the TI-time then recovery of two-dimensional inversion-recovery gradient-echo sequence and a PSIR-sequence on a complete set of short-axis sections (encompassing the entire LV) and three long-axis sections (four-, three- and two-chamber views). LE images were acquired by using 8-mm section thickness. Presence of at least 1 focal lesion with nonischemic regional distribution in inversion recovery-prepared gadolinium-enhanced T1-weighted images was considered a positive criterion.

Data were analyzed using software for evaluating CMR images (cmr^42^, Circle Cardiovascular Imaging Inc., Calgary, Canada).

All patients received a complete pulmonary function test (PFT) according to international recommendations [15]–[17]. The measurements were obtained by using a MasterScreen® Body/Diffusion (CareFusion, Hoechberg, Germany). All collected PFT-parameters were expressed as a percentage of the predicted values, which were obtained from regression equations by Quanjer et al [18] and Cotes et al [19].

CPET was carried out on an electromagnetically braked cycle ergometer (Excalibur Sport, Lode B.V. Groningen, The Netherlands) with respiratory gas exchange analysis on a breath-by-breath basis (MasterScreen® CPX, CareFusion, Hoechberg, Germany) and additional blood gas analysis according to a standardized procedure [20]. The used reference values for peak VO_2_/kg (ml/min/kg) are based on the recommendations of Cooper et al. (women: VO_2_ max/kg  = 42.83– (0.371 * years); men: VO_2_ max/kg  = 50.02– (0.394 * years)) [21].

OUES was computed by a linear least squares regression from the oxygen uptake (VO_2_) on the logarithm of the minute ventilation (VE) according to the following equation: VO_2_  =  a * log_10_VE + b. Constant ‘a’ is called the OUES, as it represents the rate of absolute increase in oxygen uptake in response to a change in minute ventilation. To compare the OUES results from our study group with reference values, we estimated the predicted OUES for age, body surface area (BSA) and sex-matched normal participants according to the equations published by Hollenberg et al. (for women: OUES  = 1175–15.8 * age +841 * BSA; for men: OUES  = 1320–26.7 * age +1394 * BSA) [22].

Data were analyzed using IBM^®^ SPSS^®^ Statistics 19 for Windows^®^ computer software. All values were reported as mean ± standard deviation except when specified otherwise. Unpaired Student's t-test and Wilcoxon signed-rang-test were used for appropriate comparisons. Relationships between variables were assessed by the Pearson's correlation coefficient (r). The receiver operating characteristic (ROC) curve method was used to plot the true positive rate (sensitivity) in function of the false positive rate (1-specificity) for different cut-off points of predicted OUES values.

To test the simultaneous influence of other independent predictor variables on the dependent target variable (cardiac sarcoidosis) and to reduce the risk of overfitting the data we used a binary logistic regression model with stepwise forward selection (*p_in_<0.05; p_out_>0.1*). The following candidate variables were used: radiographic stage (Typ 0 through Typ IV), FEV_1_ (absolute value in L), DLCO/VA (%pred), LVEF (%),VO_2_max (absolute value in ml/min/kg), OUES (absolute value in ml/min/log(ml/min)). This analysis was repeated with the cut-off value identified in the ROC-analyses in order to yield maximal clinical applicability. Results of the multivariable analysis are reported as β-coefficient, 95% confidence intervals and p-value. A *p*-value of less than 0.05 was reported as significant for all analyses.

## Results

A total of 37 patients (mean age 44.9±13.8 years; 11 female) was assessed. Demographic, clinical and diagnostic findings for the groups with cardiac sarcoidosis (11/37, 30%) and without cardiac sarcoidosis are shown in Tables 2 and 3. Age, gender, height, weight, Body Surface Area and Body Mass Index did not differ significantly between the two groups. Only the radiographic distribution of pulmonary stages was unevenly distributed with more advanced stages among patients with cardiac involvement (*p = 0.031*).

**Table 2:** Summary of Demographic, CMR-, Pulmonary function tests and CPET-Data (n = 37).

	Group with Cardiac Sarcoidosis	Group without Cardiac Sarcoidosis	p-value
	(n = 11)	(n = 26)	
Mean age [yrs]	44.3±14.6	45.1±13.7	NS
Pulmonary stage (0/I/II/III/IV)	(0/1/4/3/3)	(1/5/18/1/1)	0.031‡
height [cm]	177.5±12.6	175.7±10.8	NS
weight [kg]	82.4±15.4	84.7±15.2	NS
BMI [kg/m2]	26.0±3.2	27.5±4.8	NS
BSA [m2]	1.99±0.24	2.00±0.21	NS
**CMR-findings**	**(n = 10)**	**(n = 26)**	
Myocardial EGE (SI)	4.4 (3.0–22.2)	2.9 (1.2–6.6)	0.006‡
T2 signal intensity (SI)	2.0 (1.6–2.7)	1.7 (1.2–2.0)	0.013§
LGE (n)	6/10 (60%)	0/26 (0%)	<0.0001‡
Abnormal wall motion (n)	6/10 (60%)	2/26 (7.7%)	<0.0001‡
Mean LVEF [%]	56±9	67±6	<0.0001§
Pericardial effusion (n)	2/10 (20%)	2/26 (7.7%)	NS
**Pulmonary function tests**	**(n = 11)**	**(n = 26)**	
FEV1 (%pred)	78.7±16.4	86.9±19.8	NS
VC (%pred)	77.1±21.9	89.7±13.5	NS
TLC (%pred)	88.4±18.8	100.8±14.8	NS
DLCO (%pred)	61.1±23.6	78.0±18.0	NS
CPET	(n = 11)	(n = 26)	
Peak VO2 abs. [ml/min/kg]	18.3±5.9	23.7±5.1	0.014§
Peak VO2 (%pred)*	55.9±21.2	73.9±16.0	<0.005§
RER max.	1.19±0.14	1.12±0.08	NS
AaDO2 max. [mmHg]	38.9±12.5	35.1±10.8	NS
OUES abs. [l/min/log(l/min)]	1669.70±635.82	2392.43±809.25	0.013§
OUES (%pred)†	59.3±19.1	88.0±15.4	<0.0001§

BMI  =  body mass index; BSA  =  body surface area; CMR  =  cardiovascular magnetic resonance; LVEF  =  left ventricular ejection fraction; CPET  =  cardiopulmonary exercise testing; EGE  =  Early gadolinium enhancement; LGE  =  Late gadolinium enhancement.

*Reference values according to equations published by Cooper et al. [21]

† Reference values according to equations published by Hollenberg et al [22].

‡ Wilcoxon signed-rang-test.

§ Unpaired Student's t-test.

**Table 3:** Eleven patients with evidence for Cardiac Sarcoidosis (CS).

Patient #	Age (yrs)/Sex	Chest x-ray	CMR-criteria (tissue marker)	T2-STIR/EGE/LGE	ECG	Decreased LVEF/abnormal wall motion	LVEF	Pericardial effusion	OUES	OUES
		(Pulmonary stage)					[%]		[ml/min/log]	[%pred.]
1	46/w	4	(2/3)	no/yes/yes	normal	no/yes	54	no	855.79	47
2*	51/m	4	(1/3)	no/no/yes	1st AV block, PVC	yes/yes	45	no	900.70	35
3	61/m	3	(3/3)	yes/yes/yes	left-axis deviation	no/no	61	no	2381.76	99
4*	71/m	4	(1/3)	no/yes/no	complete RBBB	yes/yes	43	no	1436.38	57
5	30/m	1	(2/3)	yes/yes/no	normal	no/yes	61	yes	1521.59	45
6	51/w	3	(2/3)	no/yes/yes	normal	no/no	65	no	964.73	56
7*	33/m	2	(1/3)	no/yes/no	pathological Q	yes/yes	60	no	1336.43	40
8	47/m	2	(2/3)	no/yes/yes	LBBB	no/no	67	no	2372.60	82
9	45/m	3	(1/3)	no/no/yes	normal	yes/yes	49	yes	2335.01	67
10	22/m	2	(2/3)	yes/yes/no	normal	yes/yes	54	no	1787.81	53
11†	30/m	2	no CMR	no CMR	2nd AV block, PVC, left-axis deviation	no/yes	no CMR	no	2473.88	71

CMR  =  Cardiovascular Magnetic Resonance; EGE  =  Early myocardial Gadolinium Enhancement; LGE  =  Late myocardial Gadolinium Enhancement; LVEF  =  Left Ventricular Ejection Fraction; ECG  =  Electrocardiogram; OUES  =  oxygen uptake efficiency slope; RBBB  =  right bundle branch block; LBBB  =  left bundle branch block; PVC  =  premature ventricular contraction.

*Coronary angiography excluded significant obstructive coronary artery disease.

† no CMR because of implanted pacemaker.

Only 1/37 patients (3%) could not be examined by CMR during the study because he presented a symptomatic 2nd degree AV block (Mobitz II), and a pacemaker had to be implanted. However, in this case a transthoracic echocardiography was performed and showed regional wall motion abnormalities with normal LV-function and exclusion of pericardial effusion, so that the patient was not excluded from the study. Of the other 36 patients, 4/36 (11%) had pericardial effusion, 9/36 (25%) patients had increased global myocardial early gadolinium enhancement (EGE) ratio in T1-weighted images, 8/36 (22%) patients had regional or global myocardial SI increase in T2-weighted images and 6/36 (17%) had local myocyte injury and/or scar, confirmed by myocardial late gadolinium enhancement (LGE) with typical nonischemic regional distribution [23].

In 5 patients (14%) cardiac involvement was diagnosed according to revised guidelines of diagnosing cardiac sarcoidosis from the Japanese Society of Sarcoidosis and Other Granulomatous Disorders including CMR-findings (Tab. 1) [7]. The remaining 6 patients (17%) of the cardiac sarcoidosis group presented at least two of the three tissue-based CMR-findings for myocardial inflammation and fulfilled the proposed diagnostic CMR criteria (i.e., Lake Louise Consensus Criteria) for myocarditis [14]. It is likely that abnormalities on CMR in these patients represent cardiac sarcoidosis even in the absence of clinical cardiac abnormalities[24].

Cardiac function (LVEF) was mildly impaired on average, but significantly poorer in the patient group with cardiac sarcoidosis compared to the group without cardiac sarcoidosis due to the sarcoidosis associated heart failure after exclusion of other possible causes (57±7 vs 68±5%, *p<0.0001*). Nevertheless, both groups were in the range of preserved ejection fraction.

No significant differences in the preserved lung function parameters between the two groups (CS-group vs non-CS-group) were observed (Tab. 2). 10/37 (27%) patients presented a ventilatory dysfunction as defined by ATS/ETS guidelines [17] (4/11, 36% in CS-group vs 6/26, 23% in non-CS-group, *p = 0.544*). 22/37 (59%) patients showed a decreased transfer factor (i.e., DLCO corrected for Hb <80% of predicted; 8/11, 73% in CS-group vs 14/26, 54% in non-CS-group, *p = 0.384*).

All patients completed the cardiopulmonary exercise test (CPET) without any complication. Mean peak respiratory exchange ratio (RER) was 1.14±0.11. Only two patients stopped the exercise test before reaching the metabolic exertion (i.e., peak RER <1.0) due to pulmonary limitations (1/11, 9% in CS-group vs 1/26, 4% in non-CS-group, *p = 0.806*).

**Figure 1:**
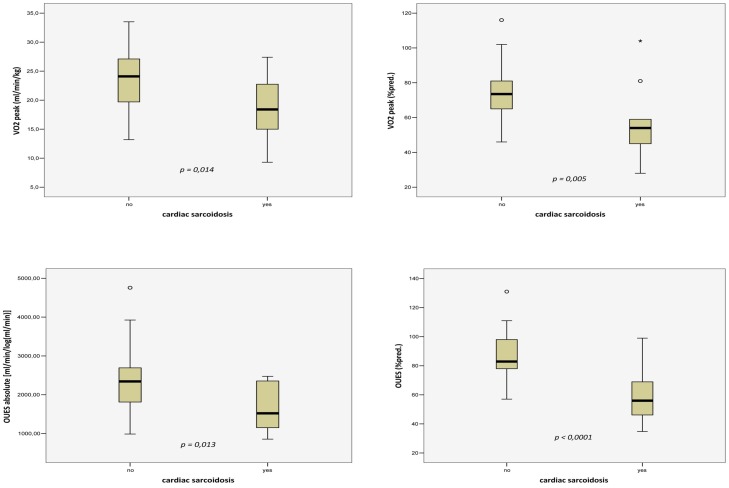
Comparison of CPET parameters in patients with and without cardiac sarcoidosis (CS). Peak VO_2_ [ml/min/kg] upper left, peak VO_2_ [% of predicted] upper right, OUES [ml/min/log(ml/min)] lower left, and OUES (% of predicted) lower right panel. All panels: Outliers (values that are between 1.5 and 3 times the interquartile range) are represented by circles beyond the whiskers. Extreme values (values that are more than 3 times the interquartile range) are represented by asterisk beyond the whiskers. P-values are given for the univariable comparison.

Mean peak VO_2_ values were 22.1±5.8 and 68.5±19.3 when expressed respectively in ml/min/kg and as percent of predicted value. In comparison to the patient group without cardiac sarcoidosis the patients with cardiac involvement had lower peak VO_2_-values (18.3±5.9 vs 23.7±5.1 ml/min/kg, *p = 0.014*) and significantly lower percentage of predicted peak VO_2_-values (55.9±21.2 vs 73.9±16.0%pred., *p = 0.005*) (Figure 1 upper left and right panel).

Mean maximal AaDO_2_ was 36.2±11.3 mmHg and showed no significant difference between both groups (38.9±12.5 vs 35.1±10.8 mmHg, *p = 0.358*).

In the entire study population, OUES could be calculated and ranged from 855.79 to 4756.39 [ml/min/log(ml/min)] (mean ± SD: 2177.56±824.15). Absolute OUES values and percentage of predicted OUES values according to the equations released by Hollenberg et al. were significantly poorer in the patient group with evidence of cardiac sarcoidosis (1669.70±635.82 vs 2392.43±809.25 [ml/min/log(ml/min)], *p = 0.013* and 59.3±19.1 vs 88.0±15.4%pred., *p<0.0001*, respectively) (Figure 1, lower left and right panel).

When related to the resting cardiac function, LVEF showed a positive correlation with predicted peak VO_2_ values (*r = 0.414, p = 0.012*). Moreover, LVEF was significantly and directly related to predicted OUES values (*r = 0.443, p = 0.007*).

**Figure 2:**
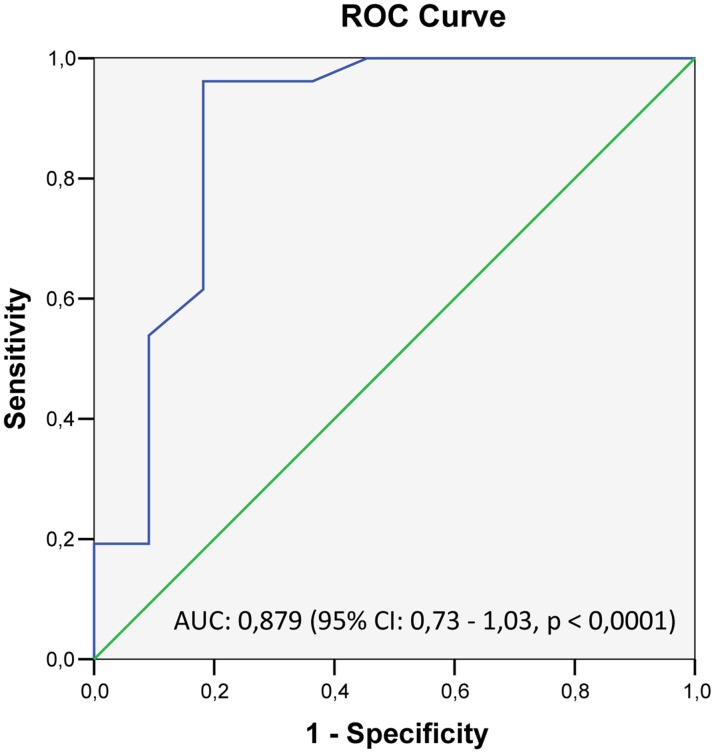
The Receiver Operating Characteristic (ROC) curve of different OUES cut-off points (%pred) and CS diagnosis. A OUES <70% of predicted as cut-off point had the largest diagnostic discriminatory power for diagnosing cardiac sarcoidosis.

In order to assess and compare operative characteristics of OUES for the distinctions between patients with and without cardiac sarcoidosis, ROC-curves were plotted. The area under curve (AUC) was 0.879 (95% CI: 0.73 to 1.03, *p<0.0001*) (Figure 2). 70% of predicted OUES as cut-off point had the largest diagnostic discriminatory power for diagnosing cardiac sarcoidosis (96% sensitivity, 82% specificity and 89% overall accuracy) (Table 4).

**Table 4:** Association between observed and predicted cardiac sarcoidosis according to the best OUES cut-off point at 70% of predicted.

	Predicted			
	*No Cardiac sarcoidosis*	*Cardiac sarcoidosis*	*Total*	*Percentage*
	(n = OUES >70%pred)	(n = OUES <70%pred)	(n)	correct (%)
**Observed**				
*No cardiac sarcoidosis (n)*	25 (true negative)	1 (false positive)	26	96.2
*Cardiac sarcoidosis (n)*	3 (false negative)	8 (true positive)	11	72.7
*Overall correct (%)*				89.2

### Multivariable Analyses

When all candidate variables were included in the regression model only OUES and LVEF were significantly associated with the presence of cardiac sarcoidosis (Table 5). When the cut off point (70% of predicted) for OUES was used in the model, it returned as the strongest predictor of cardiac sarcoidosis (Odds ratio: 100.43, 95% CI: 1.99 to 5064, *p<0.001*). LVEF remained as a clinical predictor (Odds ratio: 0.776, 95% CI: 0.578 to 1.042, *p = 0.092*) in both models, but contributed little improvement (correct classified with OUES (70%pred) 91.7%, adding LVEF 94.4%).

**Table 5:** Results of the multivariable analyses (stepwise forward p_in_<0.05; p_out_>0.1).

Variable	Values	β-coefficient	95% Confidence interval	p-value
Continuous Values				
OUES	% predicted	0.942	0.886–1.01	0.056
LVEF	continuous	0.751	0.591–0.944	0.014
radiographic stage	Stage 0–4	Excluded from the model	-	0.234
FEV1	continuous	Excluded from the model	-	0.326
VO2max	continuous	Excluded from the model	-	0.069
DLCO/VA	% predicted	Excluded from the model	-	0.206
**Categorical Values**				
OUES	categorical	100.434	1.992–5064	0.021
	(cut-off 70% pred)			
LVEF	continuous	0.776	0.578–1.042	0.092
radiographic stage	Stage 0–4	Excluded from the model	-	0.215
FEV1	continuous	Excluded from the model	-	0.911
VO2max	continuous	Excluded from the model	-	0.871
DLCO/VA	% predicted	Excluded from the model	-	0.807

Two models were calculated with either OUES in continuous (upper table) or as categorical values (70% predicted, lower table).

Radiographic findings (i.e., pulmonary stage of sarcoidosis according to chest x-ray) did not fulfill inclusion criteria of this method. In addition, neither PFT-parameters associated with ventilatory impairment nor PFT-parameters associated with gas exchange disorder qualified for the regression model with cardiac sarcoidosis as the dependent variable (p_in_ <0.05).

## Discussion

The main findings of the present study were: (1) cardiac sarcoidosis is associated with a poor cardiovascular response to exercise and can be verified by CPET. (2) Patients with cardiac sarcoidosis had significantly lower levels of OUES due to the sarcoidosis associated heart failure. (3) A cut-off point of OUES (70% of predicted according to equations published by Hollenberg et al) was able to discriminate between patients with and without cardiac sarcoidosis (Odds ratio: 100.43, 95% CI: 1.99 to 5064, *p<0.001*).

In order to identify and treat patients with cardiac involvement in histologically proven sarcoidosis at an early stage a screening-series of noninvasive diagnostic approaches is recommended. However, the incidence of cardiac involvement is much higher when specific cardiac tests are performed in unselected patients with sarcoidosis [25]–[27]. This finding underscores the fact, that patients with cardiac sarcoidosis are often asymptomatic and a potentially dangerous cardiac involvement may remain undetected. Several studies showed that cardiovascular magnetic resonance (CMR) imaging improves the sensitivity and specificity of diagnosis of cardiac sarcoidosis [14], [28]–[30]. Late gadolinium enhancement has emerged as the dominant CMR sequence for evaluation of cardiac sarcoidosis [28]–[30]. Preliminary observations suggest that monitoring gadolinium enhancement may also be helpful in the assessment of the efficacy of steroid therapy [11]. Patel et al. compared late gadolinium enhancement CMR with Japanese Ministry of Health and Welfare (JMH) criteria in a series of 81 patients with biopsy proven extracardiac sarcoidosis [30]. In this study late gadolinium enhancement was more than twice as sensitive for CS as JMH criteria.

In order to assess best diagnostic accuracy of CS, revised Japanese guidelines including late gadolinium enhancement CMR and the CMR criteria according to the International Consensus Group on CMR Diagnosis of Myocarditis were applied in our study [7], [14].

The present study investigated the usefulness of CPET with calculation of OUES for the evaluation of patients with sarcoidosis in a setting that could become part of a routine screening.

In line with previous studies, we found that cardiac function (i.e., LVEF) was closely related to OUES [31], [32]. Interestingly, we also demonstrated that in patients with sarcoidosis cardiac involvement was associated with a poor cardiovascular exercise performance, both in terms of oxygen uptake (peak VO_2_) and oxygen uptake efficiency slope (OUES). Peak VO_2_ is strongly influenced by the motivation of the patient, the selected exercise protocol and the tester's subjective choice of the test end point [33]. In contrast, the OUES is an objective and reproducible measure of cardiopulmonary function reserve that can also be measured with submaximal exercise [34]. Therefore, OUES is a better parameter of CPET in order to evaluate patients with sarcoidosis and should be calculated and recorded with peak VO_2_.

In the present study, we found that evidence for sarcoid heart failure was associated with decreased levels of oxygen uptake efficiency during exercise. The chance to diagnose cardiac sarcoidosis in patients with an OUES <70% of predicted was 67-times higher than in patients with an OUES >70% of predicted. In addition, only OUES remained in the multiple regression model with cardiac sarcoidosis as the dependent variable, when possible confounders were offered in the statistical model. Therefore these findings strongly suggest that a reduced cardiovascular performance measured by OUES is associated with cardiac sarcoidosis.

In the interpretation of our study results, some possible limitations have to be discussed. First, the number of included patients was limited and our results should be prospectively confirmed in a larger cohort, before CPET and OUES is introduced into the assessment routine of patients with sarcoidosis. However, the predictive power of OUES for results of CMR was strong (OR 67) and plausible which makes controversial future findings unlikely. Secondly, we cannot rule out that the degree of physical fitness and the extent of pulmonary impairment may also affect the relationship between oxygen uptake and ventilation during exercise in individual patients with sarcoidosis [35]. For example, the false positive tested patient in our study showed a moderate restrictive ventilatory impairment and a significant gas exchange disorder in rest and during exercise. Third, we did not analyze complete medication history and future longitudinal studies are needed to determine whether a therapy with corticosteroids or other immunosuppressants is also associated with an increase of OUES in patients with cardiac sarcoidosis. The effect of drug intervention could be a plausible reason for the false negative findings in our study. But we felt that the diagnosis of cardiac sarcoidosis was only firmly established when patients with less than three CMR-criteria also fulfilled the Japanese Society Criteria. The may have biased the results towards sicker patients. However, we wanted to focus on patients with possible clinical consequences, such as high dose corticosteroid treatment. On one hand, we could therefore include the patient who had already cared for with a cardiac pacemaker and thus clinically cardiac sarcoidosis. On the other hand, the absence of significant functional cardiac disorders could explain the elevated OUES values in the false negative tested patients. It is well known that the clinical manifestations of cardiac sarcoidosis depend upon the location and the extent of the myocardium involved [3]. Roberts et al. reported the result of 113 necropsy patients with cardiac sarcoidosis. They found that the left ventricular free wall is the most common location for granulomas and scars (96%), followed by the intra-ventricular septum (73%). Cardiac infiltration by sarcoid granulomas in these locations may cause diminished systolic contractile function or diastolic dysfunction more frequently.

The diagnosis of cardiac involvement through MRI was limited due to the retrospective nature of this study. This was possibly the absence of a complete stack of short axis images to calculate LV volumes and function and the relative high slice thickness (15 mm) of STIR and EGE images, with consequent low spatial resolution, compared to the current gold standard (6–10 mm, as used for LGE).

Furthermore, recent research pointed out the importance of biomarkers for the prognosis of patients with cardiac involvement of sarcoidosis. Therefore the inclusion of e.g. plasma NT-proBNP [36], high-sensitive cardiac troponin T [37], and NT-proatrial natriuretic peptide [38] in future prospective studies may improve the results.

## Conclusion

In conclusion, cardiopulmonary exercise test (CPET) potentially improves detection of cardiac sarcoidosis and should be implemented in the routine evaluation of patients with histologically proven sarcoidosis. Low OUES (<70%pred.), was the single best predictor of cardiac sarcoidosis in our study. Therefore, OUES should be assessed at each examination, also in submaximal tests. Future studies need to confirm OUES as a clinically useful and powerful predictor of cardiac sarcoidosis. In addition, the role of OUES in monitoring of drug-treatment effects in patients with cardiac sarcoidosis will be subject to further investigations.
